# Investigating therapeutic response to netarsudil in glaucoma subjects with the *ARHGEF12* risk variant

**DOI:** 10.3389/fphar.2026.1803432

**Published:** 2026-05-08

**Authors:** Anusha Mamidipaka, Isabel Di Rosa, Marine-Ayan Ibrahim Aibo, Fangming Jin, Rebecca Salowe, Roy Lee, Mina Halimitabrizi, Leila Ghaffari, Victoria Addis, Laxmi Moksha, Gui-Shuang Ying, Joan M. O’Brien

**Affiliations:** 1 Department of Ophthalmology, Center for Genetics of Complex Disease, Scheie Eye Institute, University of Pennsylvania, Philadelphia, PA, United States; 2 Center for Preventive Ophthalmology and Biostatistics, Perelman School of Medicine at the University of Pennsylvania, Philadelphia, PA, United States

**Keywords:** ARHGEF12, netarsudil, neuroprotection, primary open-angle glaucoma, Rho kinase (ROCK) inhibitor, Rho/ROCK pathway, Rhopressa

## Abstract

**Introduction:**

*ARHGEF12* variants, which regulate the RhoA/ROCK pathway, are associated with primary open-angle glaucoma (POAG) in individuals of African ancestry and may influence response to Rho-kinase inhibitors such as netarsudil.

**Methods:**

We analyzed 1,844 African ancestry subjects with POAG, comparing baseline demographic and ocular phenotypes across *ARHGEF12* genotype groups (non-carriers, heterozygous carriers, and homozygous carriers). Netarsudil response was evaluated in a subset of 66 subjects (114 eyes). Generalized estimating equations were used to compare the change in intraocular pressure (IOP) and other ocular measures from baseline to 12 months across the *ARHGEF12* genotype groups.

**Results:**

Baseline glaucoma severity and ocular phenotypes including IOP were similar across all *ARHGEF12* genotype groups (all *p* > 0.05). Among netarsudil-treated eyes, mean IOP reduction at 12 months was −1.77 ± 5.06 mmHg (SD) in non-carriers and 1.58 ± 6.25 mmHg (SD) in combined heterozygous/homozygous carriers. A ≥20% IOP reduction at 12 months was achieved in 29.8% of non-carrier eyes and 34.6% of variant-carrier eyes (aOR 1.31, 95% CI 0.42–4.10, *p* = 0.64). Among the netarsudil treated eyes, no statistically significant differences were observed across genotype groups for other ocular measures, including cup-to-disc ratio, retinal nerve fiber layer thickness, and visual field mean deviation.

**Discussion:**

Despite a strong biological rationale linking *ARHGEF12* to Rho/ROCK signaling and aqueous humor outflow, these preliminary findings suggest that *ARHGEF12* variant status does not strongly predict netarsudil response, suggesting that variability in ROCK inhibitor efficacy likely reflects multifactorial and pathway-level influences beyond single-gene variation. These results should therefore be considered exploratory. Future studies with larger, treatment-naïve cohorts are needed to evaluate pharmacogenetic associations.

## 1 Introduction

Glaucoma is the global leading cause of irreversible blindness in adults and is characterized by progressive optic nerve damage, typically beginning with peripheral vision loss (Steinmetz et al., 2021). Primary open-angle glaucoma (POAG) is the most common form of the disease and arises from a complex interaction of environment and genetic factors, which remain incompletely understood (Janssen et al., 2013). Individuals of African ancestry are at higher risk of POAG development, earlier onset, and more severe disease (Tielsch, 1991; Acuff et al., 2023). In a recent large, multi-cohort GWAS study including over 11,000 subjects of African ancestry, our team identified one previously described risk allele mapping to *ARHGEF12* (*rs11824032)* and two novel candidate variants in the DBF4P2 and ROCK1P1 genes (Verma, Gudiseva, Venkata R.M. Chavali, et al., 2024).

*ROCK1P1* is an inactive partial duplication, or pseudogene, of *ROCK1*, a downstream effector of the Rho GTPase (Verma, Gudiseva, Venkata R.M. Chavali, et al., 2024). *ARHGEF12* encodes Rho guanine nucleotide exchange factor 12, or Rho GEF12, and is proposed to be a downstream effector of Rho (Fukata et al., 2001; Kourlas et al., 2000). Alterations in trabecular meshwork and Schlemm’s canal cells contribute to increased resistance to aqueous humor drainage as a result of RhoA activation (Kumar and Epstein, 2011; Springelkamp et al., 2015). This leads to elevated intraocular pressure (IOP), a risk factor for most patients with POAG (Springelkamp et al., 2015).

Inhibition of the ROCK signaling pathway has been shown to lower IOP, primarily by increasing aqueous humor outflow (Inoue et al., 2022). ROCK inhibitors, such as netarsudil (brand name *Rhopressa*), represent a relatively new class of medication in the treatment of glaucoma, having been approved for use in the US by the FDA in 2017. Of the three likely pathological variants that the GWAS identified, two are associated with the Rho/Rho-kinase pathway. Among these, the *ARHGEF12* variant was prioritized for investigation due to its upstream regulatory role and the stronger functional evidence seen in ocular tissue from our laboratory research. Given this context, it is of great interest to examine the effect of Rho-kinase inhibitors on African ancestry subjects with the *ARHGEF12* variant.

This study aims to 1) characterize the demographic and ocular phenotype profiles of POAG subjects with and without the *ARHGEF12* variant, and 2) evaluate the effect of netarsudil on IOP and other ocular phenotypes in POAG subjects with and without this variant.

## 2 Methods

### 2.1 Study population

For this study, we included POAG cases with available genotyping data for the *ARHGEF12* and *ROCK1P1* locus. The Primary Open-Angle African Ancestry Glaucoma Genetics (POAAGG) study is a large genetic study of individuals of African ancestry aimed at identifying genetic factors contributing to primary open-angle glaucoma (Charlson et al., 2015). Subjects in the POAAGG study population were over age 35 and self-identified as being of African ancestry (Black, African American, or Afro-Caribbean). All subjects underwent clinical evaluation by a glaucoma specialist or ophthalmologist and were classified as POAG cases, POAG suspects, or controls using predefined diagnostic criteria. Details on the study design and eligibility criteria have been published previously (Charlson et al., 2015). This study was approved by the University of Pennsylvania Institutional Review Board in accordance with local legislation and institutional requirements. All participants provided their written informed consent to participate in accordance with the Declaration of Helsinki.

### 2.2 Demographic, ocular phenotype, and clinical data extraction

At enrollment, subjects underwent an onsite interview and comprehensive examination. Demographic variables collected included: age, sex, body mass index (BMI), history of diabetes mellitus, tobacco and alcohol use, and family history of glaucoma. Glaucoma history included disease severity and history of surgical glaucoma interventions. Electronic health records were reviewed to supplement clinical examination findings and to ascertain prior glaucoma-related surgical history.

Ocular phenotypes measured at enrollment and follow-up appointments included central corneal thickness (CCT, µm), intraocular pressure (IOP, mmHg), cup-to-disc ratio (CDR), retinal nerve fiber layer thickness (RNFL, µm), visual field mean deviation (MD, dB), pattern standard deviation (PSD, dB), and best-corrected visual acuity (VA, LogMAR). Standardized protocols for these ocular measurements were applied during clinical examinations.

### 2.3 Netarsudil exposure definition

Subjects were classified as netarsudil (*Rhopressa*) users if they had documented cumulative use of the medication for at least 3 months. Medication adherence could not be directly verified; instead, medication exposure, laterality (left, right, or both eyes), and treatment duration were determined through clinical records. A subset of subjects received netarsudil to one eye rather than bilaterally, so only treated eyes were included in the analysis.

Baseline measurements were defined as the most recent ocular assessments obtained within 6 months prior to netarsudil initiation; values from multiple visits were not averaged. No washout period occurred, and subjects continued their existing glaucoma medications during netarsudil initiation. The final on-treatment measurement was defined as the assessment closest to 12 months after treatment initiation. Values were included only if they were recorded ± 6 months of the 12-month time point. The treatment duration eligibility criteria was selected at 12 months based on previous retrospective studies of ripasudil and other ROCK inhibitors that demonstrated stabilization of IOP reduction between approximately 6 weeks and 3 months of therapy (Wu et al., 2022). Successful netarsudil treatment was defined as a ≥20% reduction in IOP, consistent with prior studies (Gedde et al., 2021; Kass, 2002; Fahy et al., 2024).

### 2.4 Genotyping and variant classification

Genotype data for *ARHGEF12* were obtained from previously generated high-density genotyping arrays utilized within the POAAGG study (Verma, Gudiseva, Venkata R.M. Chavali, et al., 2024).

Variant calling and quality control procedures followed established pipelines, including checks for sample and SNP call rate, Hardy-Weinberg equilibrium, and concordance with previously known allele frequencies in African ancestry populations. Variants within the Rho/ROCK signaling pathway, including loci at ARHGEF12 and ROCK1P1, were identified based on prior genome-wide association analyses (Verma, Gudiseva, Venkata R.M. Chavali, et al., 2024).

Subjects were classified based on the *ARHGEF12 rs11824032* locus as non-carriers (0 variant alleles), heterozygous carriers (1 variant allele), or homozygous carriers (2 variant alleles). Genotype groups were defined as GG (homozygous wild type), GA (heterozygous), and AA (homozygous mutant).

### 2.5 Statistical analysis

The primary objective was to compare baseline demographic characteristics and ocular phenotypes between homozygous and heterozygous *ARHGEF12* variant carriers with non-carriers. Continuous variables were summarized using means and standard deviations (SD). Categorical variables were summarized using number and percentage. Categorical variables were compared using chi-square tests, with Fisher’s exact tests applied when number is small. Continuous measures were compared using analysis of variance (ANOVA); when normality assumptions were violated or significant outliers were present, the Kruskal–Wallis test was used instead. In multivariable analyses, comparison of baseline ocular phenotypes across *ARHGEF12* genotype groups were adjusted for age, sex, and ROCK1P1 variant status.

Among netarsudil users, treatment response was evaluated by assessing the change in ocular phenotypes from baseline to 12 months after treatment initiation. For baseline phenotype comparisons across *ARHGEF12* genotype groups, generalized estimating equation (GEE) models were used in place of ANOVA or Kruskal–Wallis tests to account for inter-eye correlation. For comparisons of treatment response across the three genotype groups, linear regression with GEE was used for continuous measures, and cumulative logit models with GEE were used for ordinal measures. For the binary outcome of achieving a greater than or equal to 20% reduction in IOP, a GEE logistic regression model was applied. A two-sided P-value <0.05 without correction for multiple comparisons was considered statistically significant. All analyses were conducted using SAS 9.4.

A *post hoc* power analysis was performed for comparing change in IOP from baseline to 12 months between ARHGEF12 variant–positive and variant–negative groups in user of Netarsudil. The current sample size (N = 114 eyes from 66 subjects) provided 80% power to detect a moderate effect size (0.53) at a two-sided alpha level of 0.05 when comparing ARHGEF12 variant-positive and variant-negative groups.

## 3 Results

### 3.1 Baseline demographic and ocular phenotypes by *ARHGEF12* genotype

A total of 1,844 POAG cases with *ARHGEF12* genotyping data were included, comprising 990 non-carriers, 709 heterozygous carriers, and 145 homozygous variant carriers (Supplementary Table S1). Mean age was similar across genotype groups. All other sociodemographic variables, including BMI, diabetes mellitus status, family history of glaucoma, tobacco use, and alcohol use did not significantly differ across genotype groups (Supplementary Table S1). Similarly, the groups were comparable with respect to history of glaucoma surgery, number of glaucoma medications, prevalence of severe glaucoma, and polygenic risk score (p > 0.05). Baseline ocular phenotypes were also similar across genotype groups (Table 1). Mean IOP was comparable across groups (16.75 ± 5.49 (SD), 16.39 ± 5.18, and 16.57 ± 5.50mmHg; *p* = 0.24). Similarly, retinal nerve fiber layer thickness did not significantly differ between groups (73.14 ± 14.28 (SD), 73.03 ± 14.08, 73.67 ± 13.58; *p* = 0.97).

**TABLE 1:** Phenotypes characteristics of POAG cases with and without the ARHGEF12 variant.

Statistics	ARHGEF12 non-variant carrier (Cases = 989, Eyes = 1977)	ARHGEF12 heterozygous variant carrier (Cases = 709, Eyes = 1417)	ARHGEF12 homozygous variant carrier (Cases = 145, Eyes = 290)	P-value
Central corneal thickness (CCT)				
# Of eyes	1873	1342	276	​
Mean (SD)	532.68 (39.90)	533.16 (39.19)	533.25 (38.71)	​
Univariable analysis mean difference (95% CI)	Ref	0.47 (−3.35, 4.30)	0.57 (−6.22, 7.36)	0.97
Univariable analysis mean difference (95% CI)	​	Ref	0.10 (−6.85, 7.04)	​
Multivariable analysis mean difference (95% CI)*	Ref	0.24 (−3.57, 4.04)	0.46 (−6.27, 7.19)	0.99
Multivariable analysis mean difference (95% CI)*	​	Ref	0.22 (−6.66, 7.10)	​
Intraocular pressure (IOP)				
N	1970	1412	289	​
Mean (SD)	16.75 (5.49)	16.39 (5.18)	16.57 (5.50)	​
Univariable analysis mean difference (95% CI)	Ref	−0.37 (−0.82, 0.09)	−0.18 (−1.01, 0.65)	0.30
Univariable analysis mean difference (95% CI)	​	Ref	0.18 (−0.66, 1.03)	​
Multivariable analysis mean difference (95% CI)*	Ref	−0.39 (−0.85, 0.06)	−0.21 (−1.03, 0.62)	0.24
Multivariable analysis mean difference (95% CI)*	​	Ref	0.19 (−0.66, 1.03)	​
Cup-to-disc ratio (CDR)				
N	1866	1359	276	​
Mean (SD)	0.72 (0.17)	0.72 (0.17)	0.71 (0.17)	​
Univariable analysis mean difference (95% CI)	Ref	0.01 (−0.01, 0.02)	0.00 (−0.03, 0.03)	0.77
Univariable analysis mean difference (95% CI)	​	Ref	−0.01 (−0.04, 0.02)	​
Multivariable analysis mean difference (95% CI)*	Ref	0.01 (−0.01, 0.02)	0.00 (−0.03, 0.02)	0.75
Multivariable analysis mean difference (95% CI)*	​	Ref	−0.01 (−0.04, 0.02)	​
Retinal nerve fiber layer (RNFL) thickness				
N	716	484	89	​
Mean (SD)	73.14 (14.28)	73.03 (14.08)	73.67 (13.58)	​
Univariable analysis mean difference (95% CI)	Ref	−0.11 (−2.16, 1.94)	0.53 (−3.08, 4.14)	0.94
Univariable analysis mean difference (95% CI)	​	Ref	0.64 (−3.07, 4.35)	​
Multivariable analysis mean difference (95% CI)*	Ref	−0.16 (−2.13, 1.80)	−0.37 (−4.04, 3.29)	0.97
Multivariable analysis mean difference (95% CI)*	​	Ref	−0.21 (−3.96, 3.54)	​
Visual field mean deviation (VFMD)				
N	752	575	81	​
Mean (SD)	−7.36 (8.33)	−8.27 (8.70)	−8.47 (9.48)	​
Univariable analysis mean difference (95% CI)	Ref	−0.90 (−2.05, 0.25)	−1.10 (−3.74, 1.54)	0.26
Univariable analysis mean difference (95% CI)	​	Ref	−0.20 (−2.89, 2.49)	​
Multivariable analysis mean difference (95% CI)*	Ref	−0.81 (−1.94, 0.32)	−1.17 (−3.83, 1.50)	0.30
Multivariable analysis mean difference (95% CI)*	​	Ref	−0.35 (−3.07, 2.36)	​
Pattern standard deviation (PSD)				
N	755	573	81	​
Mean (SD)	5.11 (3.47)	5.24 (3.31)	4.90 (3.16)	​
Univariable analysis mean difference (95% CI)	Ref	0.13 (−0.29, 0.55)	−0.21 (−1.07, 0.66)	0.69
Univariable analysis mean difference (95% CI)	​	Ref	−0.34 (−1.22, 0.54)	​
Multivariable analysis mean difference (95% CI)*	Ref	0.11 (−0.31, 0.53)	−0.19 (−1.07, 0.70)	0.76
Multivariable analysis mean difference (95% CI)*	​	Ref	−0.30 (−1.19, 0.60)	​
Visual acuity (logMAR)				
N	1741	1236	254	​
Mean (SD)	0.42 (0.89)	0.41 (0.89)	0.39 (0.87)	​
Univariable analysis mean difference (95% CI)	Ref	0.00 (−0.07, 0.07)	−0.03 (−0.15, 0.08)	0.86
Univariable analysis mean difference (95% CI)	​	Ref	−0.03 (−0.15, 0.09)	​
Multivariable analysis mean difference (95% CI)*	Ref	0.00 (−0.07, 0.07)	−0.03 (−0.14, 0.09)	0.89
Multivariable analysis mean difference (95% CI)*	​	Ref	−0.03 (−0.15, 0.09)	​

* Adjusted for age, sex and ROCK1P1.

### 3.2 Netarsudil treatment response by *ARHGEF12*

Based on available ocular phenotype data, a total of 66 subjects were treated with netarsudil, including 34 non-carriers (57 eyes) and 32 heterozygous/homozygous carriers (57 eyes) (Supplementary Table S2). Among treated subjects, 48 received bilateral treatment (96 eyes) and 18 received unilateral treatment (18 eyes). Baseline demographic and clinical characteristics, including age, sex, BMI, diabetes status, duration of netarsudil use, number of glaucoma medications, and glaucoma severity, did not differ significantly between genotype groups (all *p* > 0.05) (Supplementary Table S2). The proportion of eyes with severe glaucoma was comparable between groups (50.88% in non-carrier eyes vs. 64.91% in heterozygous/homozygous carrier eyes, *p* = 0.14). Similarly, the mean number of glaucoma medications was similar between non-carriers (3.48 ± 0.72) and heterozygous/homozygous carriers (3.47 ± 0.67). The mean duration of netarsudil use was 34.05 ± 26.59 months in non-carriers and 29.03 ± 23.28 months in heterozygous/homozygous carriers (*p* = 0.36).

Baseline ocular phenotypes among netarsudil-treated eyes were largely comparable between genotype groups. However, RNFL thickness was significantly lower in non-carriers compared to heterozygous/homozygous variant carriers (52.22 ± 10.11 µm vs. 64.25 ± 8.49 µm, *p* = 0.03); notably, these measurements were available in a limited subset of eyes (9 non-carrier eyes and 12 carrier eyes) (Supplementary Table S3). Mean baseline IOP was similar between groups.

Changes in ocular phenotypes from baseline to 12 months during netarsudil therapy did not differ significantly by *ARHGEF12* genotype (Table 2). Mean IOP reduction at 12 months was −1.77 ± 5.06 mmHg in non-carriers and −1.58 ± 6.25 mmHg in heterozygous/homozygous carriers (*p =* 0.68). No significant genotype-associated differences were observed for CDR, RNFL thickness, VF MD, PSD, or VA. A ≥20% reduction in IOP was achieved in 29.82% of non-carrier eyes and 34.55% of heterozygous/homozygous carrier eyes (adjusted OR 1.31; 95% CI 0.42–4.10, *p* = 0.64) (Table 3).

**TABLE 2:** Change from baseline at 12 months (± 6 months) for ocular phenotypes among Netarsudil users with and without the ARHGEF12 variant.

Statistics	ARHGEF12 non-variant carriers (Cases = 34, Eyes = 57)	ARHGEF12 heterozygous/homozygous variant carriers (Cases = 32, Eyes = 57)	P-value
Intraocular pressure (IOP)			
N	57	55	​
Mean (SD)	−1.77 (5.06)	−1.58 (6.25)	​
Univariable analysis mean difference (95% CI)	Ref	0.19 (−2.13, 2.51)	0.87
Multivariable analysis mean difference (95% CI)*	Ref	0.48 (−1.81, 2.76)	0.68
Cup-to-disc ratio (CDR)			
N	46	46	​
Mean (SD)	0.01 (0.03)	0.01 (0.03)	​
Univariable analysis mean difference (95% CI)	Ref	0.00 (−0.02, 0.01)	0.79
Multivariable analysis mean difference (95% CI)*	Ref	0.00 (−0.02, 0.01)	0.52
Retinal nerve fiber layer (RNFL) thickness#			
N	3	4	​
Mean (SD)	1.67 (2.89)	1.75 (3.30)	​
Univariable analysis mean difference (95% CI)	Ref	0.08 (−4.30, 4.47)	0.97
Visual field mean deviation (VFMD)			
N	6	12	​
Mean (SD)	−1.86 (3.12)	0.06 (4.73)	​
Univariable analysis mean difference (95% CI)	Ref	1.91 (−2.07, 5.90)	0.38
Multivariable analysis mean difference (95% CI)*	Ref	−2.63 (−6.36, 1.10)	0.15
Pattern standard deviation (PSD)#			
N	6	6	​
Mean (SD)	−0.43 (0.72)	−0.25 (1.41)	​
Univariable analysis mean difference (95% CI)	Ref	0.18 (−1.09, 1.44)	0.79
Visual acuity (logMAR)			
N	30	33	​
Mean (SD)	0.09 (0.40)	0.08 (0.22)	​
Univariable analysis mean difference (95% CI)	Ref	0.00 (−0.16, 0.16)	0.96
Multivariable analysis mean difference (95% CI)*	Ref	0.02 (−0.10, 0.13)	0.78

* Adjusted for age, sex, ROCK1P1, baseline phenotype value, glaucoma severity stage, number of glaucoma medications, history of glaucoma surgery, and diabetes status; for VFMD, ROCK1P1 is not adjusted because the small sample size of ROCK1P1 variant subjects causes convergence issue. Multivariable analysis was not conducted for PSD, and RNFL, due to the small sample size.

**TABLE 3:** IOP reduction (≥20%) in Netarsudil users with and without the ARHGEF12 variant.

Statistics	ARHGEF12 non-variant carriers (Cases = 34, Eyes = 57)	ARHGEF12 homozygous and heterozygous variant carriers (Cases = 31, Eyes = 55)	P-value
IOP reduction			
No	40 (70.18)	36 (65.45)	​
Yes	17 (29.82)	19 (34.55)	​
Univariable analysis Odds ratio (95% CI)	Ref	1.24 (0.51, 3.00)	0.63
Multivariable analysis Odds ratio (95% CI)*	Ref	1.31 (0.42, 4.10)	0.64

* Adjusted for age, sex, ROCK1P1, baseline IOP, value, glaucoma severity stage, number of glaucoma medications, history of glaucoma surgery, and diabetes status.

## 4 Discussion

Our findings indicate that the *ARHGEF12 rs11824032* variant status does not appear to strongly influence baseline glaucoma ocular phenotypes or therapeutic response to the Rho-kinase inhibitor netarsudil in African ancestry cases with POAG. Despite its role in IOP control *via* the RhoA/ROCK signaling pathway, we found that carriers and non-carriers of the *ARHGEF12* variant had similar baseline ocular phenotypes and disease severity, suggesting the variant does not confer a distinct clinical phenotypic difference in our population. In this small cohort, the change in IOP and other ocular parameters at 12 months did not differ significantly between non-carriers and variant carriers. These results are preliminary, and the majority of subjects were already receiving multiple concurrent glaucoma medications, which may attenuate observable differences and should be considered when interpreting treatment response.

**FIGURE 1:**
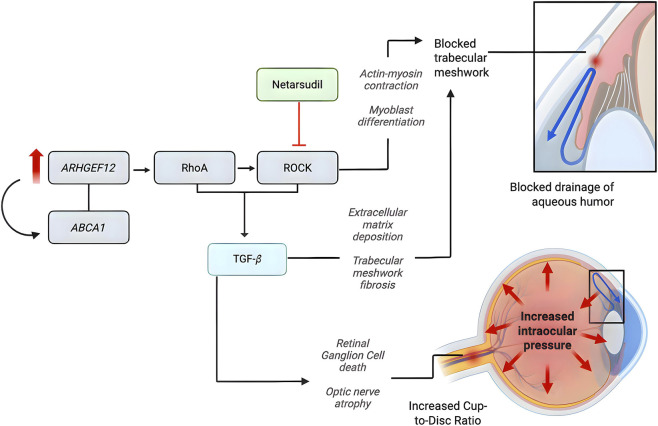
Proposed mechanism of ARHGEF12 gene and interaction with Netarsudil.

Previous literature has demonstrated an association between *ARHGEF12* and elevated IOP in European ancestry populations, as well as increased CDR in African ancestry populations (Springelkamp et al., 2015; Verma, Gudiseva, Venkata R. M. Chavali, et al., 2024). Genome-wide association studies have further implicated *ARHGEF12* in glaucoma susceptibility, with the intronic variant rs58073046 showing a robust association with increased IOP and POAG risk across large cohorts (Choquet et al., 2017; Mabuchi et al., 2017; Gharahkhani et al., 2021; Springelkamp et al., 2015). Through its signaling pathway, *ARHGEF12* activates RhoA, leading to Rho-associated kinase (ROCK) activation, increased actin-myosin contractility, myoblast differentiation, reduced trabecular meshwork and Schlemm’s canal permeability, and decreased aqueous humor outflow, ultimately elevating IOP (Inoue et al., 2022; Iglesias et al., 2015) (see Figure 1). Expression studies confirm the role of *ARHGEF12* in glaucoma-relevant ocular tissues such as the trabecular meshwork, retina, and optic nerve head (Springelkamp et al., 2015). Recent functional work from our group (Vrathasha et al., 2026) further supports biological relevance, demonstrating increased *ARHGEF12* expression in trabecular meshwork cells but decreased mRNA and protein levels in human-induced pluripotent stem cell (hiPSCs) derived retinal ganglion cells from POAG lines. In the same study, ROCK1 transcripts were significantly upregulated in trabecular meshwork cells carrying an *ARHGEF12* variant, suggesting downstream pathway perturbation, although this effect was not observed in retinal ganglion cells. However, no studies to date have directly demonstrated that the intronic variant rs58073046 or rs11824032 alters ARHGEF12 expression or RhoA/ROCK pathway activation. While the biological rationale is supported by genetic associations and pathway knowledge, this represents a critical gap, underscoring the need for functional validation to bridge population-level genetic findings with mechanistic pharmacologic effects.

Our study did not identify statistically significant differences in baseline ocular phenotypes between cases with and without *ARHGEF12* variants, suggesting that *ARHGEF12* variation alone may be insufficient to drive clinically detectable differences in disease expression. This finding indicates that IOP regulation and POAG progression likely reflect the combined effects of multiple genetic, molecular, and environmental factors, with potential compensation through parallel signaling pathways that may attenuate genotype-specific effects in clinical settings.

Rho-kinase (ROCK) inhibitors act primarily by modifying trabecular meshwork architecture to enhance aqueous humor outflow and additionally reduce aqueous humor production and episcleral venous pressure (Wu et al., 2024; Inoue et al., 2022; Lin et al., 2018; Ren et al., 2016; Berrino and Supuran, 2019). Experimental and translational studies demonstrate that ROCK inhibition promotes trabecular meshwork relaxation, increases outflow facility, and may exert anti-fibrotic and neuroprotective effects (Wiederholt et al., 1995; Rao et al., 2005; Fukunaga et al., 2009; Honjo et al., 2001; Zhang et al., 2008; Berrino and Supuran, 2019).

*ARHGEF12* variant carriers did not show a statistically significant difference in treatment response assessed by IOP; however, given the small sample size, particularly among homozygous carriers, and concurrent use of multiple glaucoma medications, these findings should be interpreted cautiously and considered preliminary. Although *ARHGEF12* lies upstream of RhoA/ROCK signaling and provides a clear biological link to netarsudil’s mechanism of action, sustained activation of this pathway is influenced by multiple regulatory inputs, including TGF-β and connective tissue growth factor-mediated fibrotic signaling (Springelkamp et al., 2015; Lessey-Morillon et al., 2014; Ren et al., 2016; Pattabiraman et al., 2014; Berrino and Supuran, 2019). Long-term ROCK inhibition with Ripasudil has been shown to induce late-onset trabecular meshwork remodeling with sustained IOP-lowering efficacy (Berrino and Supuran, 2019). This could suggest that other structural and temporal factors may be more clinically relevant than this single-variant effects in chronic POAG. These findings suggest that downstream or parallel RhoA/ROCK pathway components may dominate *in vivo*, potentially attenuating any detectable pharmacogenetic effect of *ARHGEF12* variation on netarsudil response and emphasizing the multifactorial nature of therapeutic responsiveness in POAG.

In this real-world preliminary clinical study, netarsudil was associated with small reductions in IOP, −1.77 mmHg in non-carriers and −1.58 mmHg in carriers after 12 months of use. Although a greater than or equal to 20% IOP reduction was achieved in 29.82% of non-carrier eyes, 34.55% of heterozygous/homozygous carrier eyes at 1 year, *ARHGEF12* variant status was not significantly associated with treatment response. Most subjects in our cohort had severe stage POAG and were already receiving three to four glaucoma medications at the time of netarsudil initiation, consistent with later-line use in more advanced disease, which likely limited the observable magnitude of IOP reduction and contributed to the lack of a significant genotype-treatment association. A *post hoc* power analysis using 12-month IOP change as the primary outcome indicated that our sample (N = 114 eyes) had 80% power to detect a moderate effect size (0.53) at a two-sided alpha of 0.05. While adequately powered to detect moderate or larger differences, smaller genotype-associated effects may have gone undetected. Prior studies of adjunctive Rho kinase inhibitor in patients receiving multiple IOP-lowering agents have reported small absolute reductions (−2.8 to −3.9 mmHg) and target IOP achievement in approximately half of treated eyes (Inazaki et al., 2017; Tanihara et al., 2016; 2019). In a real-world cohort of 62 eyes on maximum medical therapy, mean IOP decreased by 3.5 mmHg (−17%), with 58% achieving treatment success (Villegas and Lee, 2021). A study on a veteran cohort with advanced glaucoma on maximum therapy, mean IOP fell from 17.2 to 15.1 mmHg over 4 months, with greater than 20% reduction achieved in 30.9% of patients (Kianian et al., 2022). Pharmacologic antagonism between prostaglandin analogs and Rho kinase inhibitors (∼0.84 mmHg reduction in efficacy) may further limit netarsudil’s additive effect in heavily treated patients (Hsia et al., 2025).

This study has several limitations. The reliance on electronic health record documentation may have introduced measurement and reporting bias, particularly regarding netarsudil exposure and treatment duration. Medication adherence could not be directly verified, and patient compliance with prescribed therapy was assumed, introducing a potential source of unmeasured variability in treatment response. Retrospective and non-randomized study design also introduces potential confounding related to disease severity, prior treatments, and concurrent medication use. An additional limitation is that baseline IOP measurements were obtained from a treated clinical population. Most subjects were receiving multiple concurrent IOP-lowering medications at the time of netarsudil initiation, which may attenuate genotype-associated differences in untreated IOP; therefore, baseline values likely reflect treatment-modified rather than intrinsic physiologic IOP levels, potentially obscuring genotype-related differences present prior to therapy. Additionally, many subjects had baseline mean IOP values near target pressure (∼16–17 mmHg), introducing a potential floor effect that may have limited the observable magnitude of IOP reduction and masked genotype-specific differences. Sample size for netarsudil users was modest, limiting statistical power for detecting small genotype-specific effects. The number of homozygous *ARHGEF12* variant carriers was especially small, reducing the stability of genotype-stratified comparisons and increasing the risk of type II error. Consequently, the absence of significant differences across genotype groups should be interpreted cautiously. The study population was restricted to African ancestry subjects, which may limit generalizability to other populations. Single-variant analysis may not fully capture the polygenic or pathway-level determinants of Rho-kinase inhibitor responsiveness, and unmeasured environmental or clinical factors may also influence treatment outcomes. Finally, as this was an observational study, we did not perform polygenic or pathway analyses, so references to such mechanisms are speculative and meant only for context.

In conclusion, in this cohort of African ancestry POAG subjects, *ARHGEF12* variant status was not clearly associated with differences in baseline ocular phenotypes or IOP-lowering response to netarsudil. Although small IOP reductions were observed, substantial inter-individual variability, the small number of homozygous variant carriers, and the fact that most subjects were already receiving multiple concurrent glaucoma medications may limit the statistical power and ability to detect potential genotype-associated effects. While our preliminary findings do not provide strong evidence for single-gene variation in *ARHGEF12* prediction of ROCK inhibitor response, they highlight the need for further investigation into polygenic, pathway-level, and environmental contributors to personalized glaucoma therapy and underscore the need for larger, treatment naïve cohorts to further evaluate pharmacogenetic associations.

## Data Availability

Genotype data for POAAGG and ADAGES study participants have been deposited in the database of Genotypes and Phenotypes (dbGaP) and are publicly available as of the date of publication. Any additional information required to reanalyze the data reported in this paper is available from the lead contact upon request.

## References

[B1] Acuff K. Delavar A. Saseendrakumar B. R. Wu J.-H. Weinreb R. N. Baxter S. L. (2023). Associations between socioeconomic factors and visit adherence among patients with glaucoma in the all of Us Research Program. Ophthalmol. Glaucoma 6 (4), 405–412. 10.1016/j.ogla.2023.01.008 36746242 PMC10400726

[B2] Berrino E. Supuran C. T. (2019). Rho-Kinase inhibitors in the management of glaucoma. Expert Opin. Ther. Pat. 29 (10), 817–827. 10.1080/13543776.2019.1670812 31573364

[B3] Charlson E. S. Sankar P. S. Miller-Ellis E. Regina M. Fertig R. Salinas J. (2015). The primary open-angle African American glaucoma genetics Study. Ophthalmology 122 (4), 711–720. 10.1016/j.ophtha.2014.11.015 25576993 PMC4372490

[B4] Choquet H. Thai K. K. Yin J. Hoffmann T. J. Kvale M. N. Banda Y. (2017). A large multi-ethnic genome-wide Association Study identifies novel genetic loci for intraocular pressure. Nat. Commun. 8 (1), 2108. 10.1038/s41467-017-01913-6 29235454 PMC5727399

[B5] Fahy E. T. Montesano G. Garg A. Vickerstaff V. Konstantakopoulou E. Gazzard G. (2024). The impact of baseline intraocular pressure on initial treatment response in the LiGHT trial. Ophthalmology 131 (12), 1366–1376. 10.1016/j.ophtha.2024.06.022 38964719

[B6] Fukata Y. Kaibuchi K. Amano M. Kaibuchi K. (2001). Rho–Rho-Kinase pathway in smooth muscle contraction and cytoskeletal reorganization of non-muscle cells. Trends Pharmacol. Sci. 22 (1), 32–39. 10.1016/S0165-6147(00)01596-0 11165670

[B7] Fukunaga T. Ikesugi K. Nishio M. Sugimoto M. Sasoh M. Hidaka H. (2009). The effect of the rho-associated protein kinase inhibitor, HA-1077, in the rabbit ocular hypertension model induced by water loading. Curr. Eye Res. 34 (1), 42–47. 10.1080/02713680802531353 19172469

[B8] Gedde S. J. Lind J. T. Wright M. M. Chen P. P. Muir K. W. Vinod K. (2021). Primary open-angle Glaucoma suspect preferred practice pattern®. Ophthalmology 128 (1), P151–P192. 10.1016/j.ophtha.2020.10.023 34933743

[B9] Gharahkhani P. Jorgenson E. Hysi P. Khawaja A. P. Pendergrass S. Han X. (2021). Genome-Wide meta-analysis identifies 127 open-angle Glaucoma Loci with consistent effect across ancestries. Nat. Commun. 12 (1), 1258. 10.1038/s41467-020-20851-4 33627673 PMC7904932

[B10] Honjo M. Tanihara H. Inatani M. Kido N. Sawamura T. Yue B. Y. (2001). Effects of rho-associated protein kinase inhibitor Y-27632 on intraocular pressure and outflow facility. Investigative Ophthalmol. & Vis. Sci. 42 (1), 137–144. 11133858

[B11] Hsia Y. Wang C. Su C.-C. Huang J.-Yu Wang T.-H. Tu Y.-K. (2025). Efficacy and drug interactions of glaucoma medications: a systematic review and component network meta-analysis. Ophthalmology 132 (11), 1304–1316. 10.1016/j.ophtha.2025.07.009 40701331

[B12] Iglesias A. I. Springelkamp H. Ramdas W. D. Klaver C. C. W. Willemsen R. Van Duijn C. M. (2015). Genes, pathways, and animal models in primary open-angle glaucoma. Eye 29 (10), 1285–1298. 10.1038/eye.2015.160 26315706 PMC4815694

[B13] Inazaki H. Kobayashi S. Anzai Y. Satoh H. Inoue M. (2017). Efficacy of the additional use of Ripasudil, a rho-kinase inhibitor, in patients with glaucoma inadequately controlled under maximum medical therapy. J. Glaucoma 26 (2), 96–100. 10.1097/IJG.0000000000000552 27661993

[B14] Inoue K. Yamada S. Hoshino S. Watanabe M. Kimura K. Kamijo-Ikemori A. (2022). Glucagon-like Peptide-1 receptor agonist, liraglutide, attenuated retinal thickening in spontaneously diabetic torii fatty rats. BMC Ophthalmol. 22 (1), 206. 10.1186/s12886-022-02413-y 35524186 PMC9074190

[B15] Janssen S. F. Gorgels T. G. M. F. Ramdas W. D. Klaver C. C. W. van Duijn C. M. Jansonius N. M. (2013). The vast complexity of primary open angle glaucoma: disease genes, risks, molecular mechanisms and pathobiology. Prog. Retin. Eye Res. 37 (November), 31–67. 10.1016/j.preteyeres.2013.09.001 24055863

[B16] Kass M. A. Heuer D. K. Higginbotham E. J. Johnson C. A. Keltner J. L. Miller J. P. (2002). The ocular hypertension treatment Study: a randomized trial determines that topical ocular hypotensive medication delays or prevents the onset of primary open-angle glaucoma. Archives Ophthalmol. 120 (6), 701–713. 10.1001/archopht.120.6.701 12049574

[B17] Kianian R. Hulbert S. W. Law S. K. Giaconi J. A. (2022). Effectiveness of topical ρ-Kinase inhibitors in veterans with severe glaucoma on maximally tolerated medical therapy. Optometry Vis. Sci. Official Publ. Am. Acad. Optometry 99 (8), 626–631. 10.1097/OPX.0000000000001925 35848984

[B18] Kourlas P. J. Strout M. P. Becknell B. Veronese M. L. Croce C. M. Theil K. S. (2000). Identification of a gene at 11q23 encoding a guanine nucleotide exchange factor: evidence for its fusion with *MLL* in Acute Myeloid leukemia. Proc. Natl. Acad. Sci. 97 (5), 2145–2150. 10.1073/pnas.040569197 10681437 PMC15768

[B19] Kumar J. Epstein D. L. (2011). Rho GTPase‐mediated Cytoskeletal Organization in schlemm’s canal cells play a critical role in the regulation of aqueous humor outflow facility. J. Cell. Biochem. 112 (2), 600–606. 10.1002/jcb.22950 21268081

[B20] Lessey-Morillon E. C. Osborne L. D. Monaghan-Benson E. Guilluy C. O'Brien E. T. Superfine R. (2014). The RhoA guanine Nucleotide Exchange factor, LARG, mediates ICAM-1–Dependent mechanotransduction in endothelial cells to stimulate transendothelial migration. J. Immunol. 192 (7), 3390–3398. 10.4049/jimmunol.1302525 24585879 PMC3991232

[B21] Lin C.-W. Sherman B. Moore L. A. Laethem C. L. Lu D. W. Pattabiraman P. P. (2018). Discovery and preclinical development of netarsudil, a novel ocular hypotensive agent for the treatment of glaucoma. J. Ocular Pharmacol. Ther. 34 (1–2), 40–51. 10.1089/jop.2017.0023 28609185 PMC5963640

[B22] Mabuchi F. Mabuchi N. Sakurada Y. Yoneyama S. Kashiwagi K. Iijima H. (2017). Additive effects of genetic variants associated with intraocular pressure in primary open-angle glaucoma. PloS One 12 (8), e0183709. 10.1371/journal.pone.0183709 28832686 PMC5568337

[B23] Pattabiraman P. P. Maddala R. Rao P. V. (2014). Regulation of plasticity and fibrogenic activity of trabecular meshwork cells by rho GTPase signaling: TM cell fibrogenic activity. J. Cell. Physiology 229 (7), 927–942. 10.1002/jcp.24524 24318513 PMC3965649

[B24] Rao P. V. Deng P. F. Sasaki Y. Epstein D. L. (2005). Regulation of Myosin light chain phosphorylation in the trabecular meshwork: role in aqueous humour outflow facility. Exp. Eye Res. 80 (2), 197–206. 10.1016/j.exer.2004.08.029 15670798

[B25] Ren R. Li G. Duong Le T. Kopczynski C. Stamer W. D. Gong H. (2016). Netarsudil increases outflow facility in human eyes through multiple mechanisms. Investigative Opthalmology & Vis. Sci. 57 (14), 6197–6209. 10.1167/iovs.16-20189 27842161 PMC5114035

[B26] Springelkamp H. Iglesias A. I. Cuellar-Partida G. Amin N. Burdon K. P. van Leeuwen E. M. (2015). ARHGEF12 influences the risk of glaucoma by increasing intraocular pressure. Hum. Mol. Genet. 24 (9), 2689–2699. 10.1093/hmg/ddv027 25637523

[B27] Steinmetz J. D. Bourne R. R. A. Briant P. S. Flaxman S. R. Taylor H. R. B. Jonas J. B. (2021). Causes of blindness and vision impairment in 2020 and trends over 30 years, and prevalence of avoidable blindness in relation to VISION 2020: the right to sight: an analysis for the global burden of disease Study. Lancet Glob. Health 9 (2), e144–e160. 10.1016/S2214-109X(20)30489-7 33275949 PMC7820391

[B28] Tanihara H. Inoue T. Yamamoto T. Kuwayama Y. Abe H. Fukushima A. (2016). One‐year clinical evaluation of 0.4% ripasudil (K‐115) in patients with open‐angle glaucoma and ocular hypertension. Acta Ophthalmol. 94 (1), e26–e34. 10.1111/aos.12829 26338317

[B29] Tanihara H. Kakuda T. Sano T. Kanno T. Imada R. Shingaki W. (2019). Safety and efficacy of Ripasudil in Japanese patients with glaucoma or ocular hypertension: 3-Month interim analysis of ROCK-J, a post-marketing surveillance Study. Adv. Ther. 36 (2), 333–343. 10.1007/s12325-018-0863-1 30610614 PMC6824384

[B30] Tielsch J. M. Sommer A. Katz J. Royall R. M. Quigley H. A. Javitt J. (1991). Racial variations in the prevalence of primary open-angle Glaucoma: the Baltimore Eye Survey. JAMA 266 (3), 369–374. 10.1001/jama.1991.03470030069026 2056646

[B31] Verma S. S. Gudiseva H. V. Chavali V. R. M. Salowe R. J. Bradford Y. Guare L. (2024). A multi-cohort genome-wide Association Study in African ancestry individuals reveals risk loci for primary open-angle glaucoma. Cell 187 (2), 464–480.e10. 10.1016/j.cell.2023.12.006 38242088 PMC11844349

[B32] Villegas N. C. Lee W.-S. (2021). Effectiveness of netarsudil as an additional therapy for glaucoma in patients already on maximally tolerated medical therapy. Clin. Ophthalmol. Auckl. N.Z. 15, 4367–4372. 10.2147/OPTH.S337105 34754176 PMC8572117

[B33] Vrathasha V. Pahl M. C. Pippin J. A. Nikonov S. He J. Halimitabrizi M. (2026). “Variant-to-Gene mapping identifies *ARHGEF12* as a primary open-angle Glaucoma Effector gene operating within retinal ganglion cells.” Genomics. 10.64898/2026.02.20.707051 41929112 PMC13042018

[B34] Wiederholt M. Bielka S. Schweig F. Lütjen-Drecoll E. Lepple-Wienhues A. (1995). “Regulation of outflow rate and resistance in the perfused anterior segment of the bovine eye.” Exp. Eye Res. 61 (2): 223–234. 10.1016/S0014-4835(05)80042-9 7556486

[B35] Wu J.-H. Chang S.-N. Nishida T. Kuo B.-I. Lin J.-W. (2022). Intraocular pressure-lowering efficacy and ocular safety of rho-kinase inhibitor in glaucoma: a meta-analysis and systematic review of prospective randomized trials. Graefe’s Archive Clin. Exp. Ophthalmol. 260 (3), 937–948. 10.1007/s00417-021-05379-7 34491427

[B36] Wu J. Wei J. Chen H. Dang Y. Lei F. (2024). Rho kinase (ROCK) inhibitors for the treatment of glaucoma. Curr. Drug Targets 25 (2), 94–107. 10.2174/0113894501286195231220094646 38155465 PMC10964082

[B37] Zhang M. Maddala R. Rao P. V. (2008). Novel molecular insights into RhoA GTPase-Induced resistance to aqueous humor outflow through the trabecular meshwork. Am. J. Physiology-Cell Physiology 295 (5), C1057–C1070. 10.1152/ajpcell.00481.2007 18799648 PMC2584993

