# Supplementary material for: Investigating therapeutic response to netarsudil in glaucoma subjects with the ARHGEF12 risk variant
Source: Front Pharmacol. 2026 May 8;17:1803432. doi: 10.3389/fphar.2026.1803432 (PMC13194400; doi:10.3389/fphar.2026.1803432)
Supplement: Supplementary file 1 [file Table1.docx]

| **Supplemental Table 1 – Demographic characteristics of POAG cases with and without the ARHGEF12 variant** | | | | |
| --- | --- | --- | --- | --- |
| **Statistics** | **ARHGEF12 non-variant carrier (Cases=990)** | **ARHGEF12 heterozygous variant carrier (Cases=709)** | **ARHGEF12 homozygous variant carrier (Cases=145)** | **P-value** |
| Age (Years) | | | | |
| N | 990 | 709 | 145 | 0.33 |
| Mean (SD) | 70.59 (11.34) | 69.76 (11.85) | 69.97 (10.82) |  |
|  | | | | |
| Sex (n, %) | | | | |
| Male | 386 (38.99) | 283 (39.92) | 61 (42.07) | 0.76 |
| Female | 604 (61.01) | 426 (60.08) | 84 (57.93) |  |
|  | | | | |
| BMI | | | | |
| N | 989 | 705 | 145 | 0.80 |
| Mean (SD) | 29.81 (6.78) | 29.71 (6.37) | 30.10 (6.54) |  |
|  | | | | |
| Diabetes Mellitus (n, %) | | | | |
| No | 576 (58.30) | 431 (60.96) | 75 (51.72) | 0.11 |
| Yes | 412 (41.70) | 276 (39.04) | 70 (48.28) |  |
|  | | | | |
| Family History of Glaucoma (n, %) | | | | |
| No | 380 (41.35) | 269 (40.88) | 59 (43.70) | 0.83 |
| Yes | 539 (58.65) | 389 (59.12) | 76 (56.30) |  |
|  | | | | |
| Duration of Netarsudil use (months) | | | | |
| N | 990 | 709 | 145 | 1.00 |
| Mean (SD) | 1.35 (8.48) | 1.34 (8.03) | 1.30 (6.52) |  |
|  | | | | |
| History of glaucoma surgery (n, %) | | | | |
| No | 697 (70.76) | 502 (71.51) | 94 (65.28) | 0.32 |
| Yes | 288 (29.24) | 200 (28.49) | 50 (34.72) |  |
|  | | | | |
| Tobacco use (n, %) | | | | |
| No | 437 (44.96) | 325 (46.30) | 72 (49.66) | 0.55 |
| Yes | 535 (55.04) | 377 (53.70) | 73 (50.34) |  |
|  | | | | |
| Alcohol use (n, %) | | | | |
| No | 513 (52.83) | 376 (53.64) | 74 (51.03) | 0.84 |
| Yes | 458 (47.17) | 325 (46.36) | 71 (48.97) |  |
|  | | | | |
| Number of glaucoma medications | | | | |
| N | 184 | 550 | 112 | 0.16 |
| Mean (SD) | 2.02 (1.41) | 1.80 (1.20) | 1.81 (1.17) |  |
|  | | | | |
| Glaucoma Severity (n, %) | | | | |
| Mild | 105 (29.91) | 71 (26.79) | 19 (30.16) | 0.44 |
| Moderate | 89 (25.36) | 82 (30.94) | 21 (33.33) |  |
| Severe | 157 (44.73) | 112 (42.26) | 23 (36.51) |  |
|  | | | | |
| PRS score | | | | |
| N | 990 | 709 | 145 | 0.53 |
| Mean (SD) | 51.94 (0.79) | 51.90 (0.76) | 51.91 (0.66) |  |
