# Supplementary material for: Investigating therapeutic response to netarsudil in glaucoma subjects with the ARHGEF12 risk variant
Source: Front Pharmacol. 2026 May 8;17:1803432. doi: 10.3389/fphar.2026.1803432 (PMC13194400; doi:10.3389/fphar.2026.1803432)
Supplement: Supplementary file 2 [file Table2.docx]

| **Supplemental Table 2 – Demographic characteristics of POAG cases using Netarsudil with and without the ARHGEF12 variant** | | | |
| --- | --- | --- | --- |
| **Statistics** | **ARHGEF12 non-variant carriers (Cases=40)** | **ARHGEF12 homozygous and heterozygous variant carrier (Cases=43)** | **P-value** |
| Age (Years) | | | |
| N | 40 | 43 | 0.89 |
| Mean (SD) | 71.60 (8.96) | 71.86 (9.43) |  |
|  | | | |
| Sex (n, %) | | | |
| Male | 20 (50.00) | 20 (46.51) | 0.75 |
| Female | 20 (50.00) | 23 (53.49) |  |
|  | | | |
| BMI | | | |
| N | 40 | 43 | 0.82 |
| Mean (SD) | 29.20 (4.84) | 29.47 (5.73) |  |
|  | | | |
| Diabetes Mellitus (n, %) | | | |
| No | 25 (62.50) | 35 (81.40) | 0.055 |
| Yes | 15 (37.50) | 8 (18.60) |  |
|  | | | |
| Family History of Glaucoma (n, %) | | | |
| No | 12 (31.58) | 15 (38.46) | 0.53 |
| Yes | 26 (68.42) | 24 (61.54) |  |
|  | | | |
| Duration of Netarsudil use (months) | | | |
| N | 40 | 43 | 0.36 |
| Mean (SD) | 34.05 (26.59) | 29.03 (23.28) |  |
|  | | | |
| History of glaucoma surgery (n, %) | | | |
| No | 26 (65.00) | 23 (54.76) | 0.34 |
| Yes | 14 (35.00) | 19 (45.24) |  |
|  | | | |
| Tobacco use (n, %) | | | |
| No | 24 (60.00) | 27 (62.79) | 0.79 |
| Yes | 16 (40.00) | 16 (37.21) |  |
|  | | | |
| Alcohol use (n, %) | | | |
| No | 16 (40.00) | 22 (51.16) | 0.31 |
| Yes | 24 (60.00) | 21 (48.84) |  |
|  | | | |
| Number of glaucoma medications | | | |
| N | 40 | 43 | 0.95 |
| Mean (SD) | 3.48 (0.72) | 3.47 (0.67) |  |
|  | | | |
| PRS score | | | |
| N | 40 | 43 | 0.35 |
| Mean (SD) | 51.95 (0.84) | 51.79 (0.66) |  |
