# Supplementary material for: Investigating therapeutic response to netarsudil in glaucoma subjects with the ARHGEF12 risk variant
Source: Front Pharmacol. 2026 May 8;17:1803432. doi: 10.3389/fphar.2026.1803432 (PMC13194400; doi:10.3389/fphar.2026.1803432)
Supplement: Supplementary file 3 [file Table3.docx]

| **Supplemental Table 3 – Ocular phenotypic characteristics of POAG cases using Netarsudil with and without the ARHGEF12 variant** | | | |
| --- | --- | --- | --- |
| **Statistics** | **ARHGEF12 non-variant carriers (Cases=34, Eyes=57)** | **ARHGEF12 homozygous and heterozygous variant carriers (Cases=32, Eyes=57)** | **P-value** |
| Glaucoma Severity (n, %) | | | |
| Mild | 10 (17.54) | 3 (5.26) | 0.14 |
| Moderate | 18 (31.58) | 17 (29.82) |  |
| Severe | 29 (50.88) | 37 (64.91) |  |
|  | | | |
| Central Corneal Thickness (CCT) | | | |
| N | 55 | 55 | 0.56 |
| Mean (SD) | 533.07 (35.53) | 538.60 (40.46) |  |
|  | | | |
| Intraocular Pressure (IOP) | | | |
| N | 57 | 55 | 0.49 |
| Mean (SD) | 19.92 (5.56) | 19.06 (6.99) |  |
|  | | | |
| Cup-to-disc Ratio (CDR) | | | |
| N | 49 | 47 | 0.75 |
| Mean (SD) | 0.85 (0.13) | 0.84 (0.12) |  |
|  | | | |
| Retinal Nerve Fiber Layer (RNFL) thickness | | | |
| N | 9 | 12 | **0.03*** |
| Mean (SD) | 52.22 (10.11) | 64.25 (8.49) |  |
|  | | | |
| Visual Field Mean Deviation (VFMD) | | | |
| N | 12 | 20 | 0.39 |
| Mean (SD) | -14.20 (10.40) | -17.81 (9.43) |  |
|  | | | |
| Pattern Standard Deviation (PSD) | | | |
| N | 9 | 17 | 0.62 |
| Mean (SD) | 7.52 (3.36) | 8.29 (3.56) |  |
|  | | | |
| Visual Acuity (logMAR) | | | |
| N | 35 | 41 | 0.51 |
| Mean (SD) | 0.45 (0.59) | 0.61 (1.19) |  |
